# Mechanical Retention and Waterproof Properties of Bacterial Cellulose-Reinforced Thermoplastic Starch Biocomposites Modified with Sodium Hexametaphosphate

**DOI:** 10.3390/ma8063168

**Published:** 2015-06-02

**Authors:** Da-wei Wang, Ying-juan Xu, Xin Li, Chao-ming Huang, Kuo-shien Huang, Chuen-kai Wang, Jen-taut Yeh

**Affiliations:** 1 Hubei Collaborative Innovation Center for Advanced Organic Chemical Materials Ministry of Education, Key Laboratory for the Green Preparation and Application of Functional Materials, Faculty of Materials Science and Engineering, Hubei University, Wuhan 430062, China; E-Mails: wang_dawei@xaut.org (D.W.); xuyingjuan@live.cn (Y.X.); lixin1992624@outlook.com (X.L.); 2 School of Materials Science and Engineering, Wuhan Institute of Technology, Wuhan 430073, China; 3 Department of Materials Engineering, Kun Shan University, Tainan 710710, Taiwan; E-Mails: charming@mail.ksu.edu.tw (C.H.); ksusec@mail.ksu.edu.tw (K.H.); 4 Graduate School of Material Science and Engineering, National Taiwan University of Science and Technology, Taipei 10607, Taiwan; E-Mail: blackatlance@hotmail.com

**Keywords:** waterproof, strength retention, thermoplastic starch

## Abstract

The waterproof and strength retention properties of bacterial cellulose (BC)-reinforced thermoplastic starch (TPS) resins were successfully improved by reacting with sodium hexametaphosphate (SHMP). After modification with SHMP, the tensile strength (σ_f_) and impact strength (I_s_) values of initial and conditioned BC-reinforced TPS, modified with varying amounts of SHMP(TPS_100_BC_0.02_SHMP_x_), and their blends with poly(lactic acid)((TPS_100_BC_0.02_SHMP_x_)_7__5_PLA_25_) specimens improved significantly and reached a maximal value as SHMP content approached 10 parts per hundred parts of TPS resin (phr), while their moisture content and elongation at break (ɛ_f_) was reduced to a minimal value as SHMP contents approached 10 phr. The σ_f_, I_s_ and ɛ_f_ retention values of a (TPS_100_BC_0.02_SHMP_10_)_75_PLA_25_ specimen conditioned for 56 days are 52%, 50% and 3 times its initial σ_f_, I_s_ and ɛ_f_ values, respectively, which are 32.5 times, 8.9 times and 40% of those of a corresponding conditioned TPS_100_BC_0.02_ specimen, respectively. As evidenced by FTIR analyses of TPS_100_BC_0.02_SHMP_x_ specimens, hydroxyl groups of TPS_100_BC_0.02_ resins were successfully reacted with the phosphate groups of SHMP molecules. New melting endotherms and diffraction peaks of V_H_-type crystals were found on DSC thermograms and WAXD patterns of TPS or TPS_100_BC_0.02_ specimens conditioned for 7 days, while no new melting endotherm or diffraction peak was found for TPS_100_BC_0.02_SHMP_x_ and/or (TPS_100_BC_0.02_SHMP_x_)_75_PLA_25_ specimens conditioned for less than 14 and 28 days, respectively.

## 1. Introduction

Starch, a completely biodegradable polysaccharide, is one of the most abundant renewable resources known. Granular starch is mostly composed of linear amylose and highly branched amylopectin and can be considered a semicrystalline material [1]. The crystalline structure of starch can be disrupted by a process called gelatinization, in which starch is first mixed with water and is subsequently stirred and heated, resulting in the disruption of the crystalline structure due to the formation of hydrogen bonds between water molecules and the free hydroxyl groups of starch [2,3]. Starch cannot be considered truly thermoplastic, because its glass-rubber transition temperature (T_g_) is higher than its decomposition temperature when it has been dried. In general, the addition of plasticizers into starch is an established method for lowering the T_g_ of starch below its decomposition temperature [4,5,6,7,8,9] and converting starch into a thermoplastic starch (TPS). As TPS is one of the most promising biobased materials available for biodegradable plastic production, its importance is growing in view of the environmental problems caused by petrochemical synthetic polymers and the expected rise in the cost of petroleum-based materials [10].

However, TPS suffers from several limitations, such as poor mechanical and waterproof properties. In the quest to improve the mechanical performance of TPS-based materials, there has been increasing research interest in TPS reinforced with various available lignocellulosic fibers [11,12]. Most recently, bacterial cellulose (BC) nanofibers were reported as an efficient reinforcing additive for preparing polymeric nanocomposites [13,14,15,16,17]. The hydrophilic nature of starch causes a rapid rise in moisture content of TPS resins, and hence leads to a significant reduction in their mechanical properties if the TPS resins were not modified during their preparation processes [18,19,20,21,22]. There are three main types of crystallinity in starch as observed in the X-ray diffraction pattern [23,24,25,26]. ‘A’ and ‘B’ types of crystallinity are mainly present in cereal (e.g., maize, wheat and rice) and tuber (e.g., potato and sago) starches, respectively, while ‘C’ type crystallinity is the intermediate between A and B type crystallinity, normally found in bean and other root starches [24,25,26]. In contrast, amylose ‘V_H_’, ‘V_A_’ or ‘E_H_’ types of crystallinity are processing-induced crystallinity, which is formed during thermomechanical processing [26,27,28,29,30,31,32,33]. However, ageing of starch materials in the rubbery state occurs by retrogradation, where the starch molecules reassociate in more ordered structures, for example, by forming simple juncture points and entanglements, helices and crystal structures [34,35,36]. The rate of retrogradation and crystallization is dependent on the plasticizer content and related to the glass-transition temperature of the starch molecules. Higher amounts of plasticizer cause an increase in the mobility of the starch chains and lower the glass-transition temperature. In fact, re-crystallization of starch molecules restrains starch from practical use, because the starch easily becomes too weak to use during long-term storage, and loses use value [37].

A great deal of effort has been made to improve the waterproof properties of thermoplastic starches by substitution, esterification or acetylation of hydroxyl groups of starch molecules using organic acids or anhydrides (e.g., citric acid, succinic, maleic and phthalic anhydrides) [21,27,37,38,39] inorganic esters (e.g., trisodium trimetaphosphate), and hydroxydiethers (e.g., epichlorohydrin) [40,41,42]. Yu and coauthors [38] showed that citric acid can form stable hydrogen-bond interactions with starch and improve waterproof properties of glycerol-plasticized thermoplastic starch at high relative humidity (RH) values, although the tensile stress of thermoplastic starch specimen reduces significantly after modification by citric acid. It was reported that the hydrophobicity of TPS improved greatly when TPS was modified by prepolymers containing –NCO groups [37]. Many laboratory approaches have been taken from acetylation/esterification of starch to starch acetates, carbonilation of starch with phenyl isocyanates, isocyanate, addition of inorganic esters to starch to produce phosphate or nitrate starch esters, production of starch ethers, and hydroxy-propylation of starches via propylene oxide modification [43]. Carvalho and coauthor [41] used several reagents, *i.e.*, phenyl isocyanate, a phenol-blocked polyisocyanate, stearoyl chloride and poly(styrene-co-glycidyl methacrylate) to react with the superficial hydroxyl groups of TPS films in the medium of methylene chloride or xylene, and found that all the treatments were effective in decreasing the hydrophilic character of the TPS surfaces.

In contrast, irradiation or chemical cross-linking technologies were also used for waterproof improvement of thermoplastic starches [18,43,44,45] Jane and coauthors [46] reported that the tensile and waterproof properties of starch compounds made from starch and zein mixtures were significantly improved by crosslinking the compounds using dialdehyde. Surface of corn starch sheets was modified by cross-linking through ultraviolet (UV) irradiation by using sodium benzoate as a photosensitizer, and the results showed that surface photo-cross-linking modification significantly reduced the hydrophilic character of the starch sheet surface and enhanced the water resistance of the starch sheets [44]. The modified TPS resins with improved waterproof properties are expected to exhibit significantly improved strength retention properties during conditioning processes. However, none of the above investigations [21,27,37,38,46] has reported the resulting strength retention properties of modified TPS resins and/or the correlation with their improved waterproof properties.

In this study, waterproof and strength retention properties of BC-reinforced TPS resins were successfully improved by reacting with sodium hexametaphosphate (SHMP). By blending small amounts of poly (lactic acid) (PLA) with SHMP-modified TPS resins, their processability, waterproof and strength retention properties were significantly improved. Possible reasons for these interesting results are reported in this investigation.

## 2. Experimental

### 2.1. Materials and Sample Preparation

Tapioca starch powders, Poly (lactic acid) (PLA) 4032D resins were purchased from Eiambeng Tapioca Starch Industry Corporation, Samutprakarn, Thailand and Nature Works Company, Blair, Nebraska, USA, respectively. The waterproof properties of tapioca starches were modified by sodium hexametaphosphate (SHMP), which was purchased from Aladdin Industrial Corporation, California, USA. *Acetobacter xylinum* (BCRC 12952) was purchased from China General Microbiological Culture Collection Center, Beijing, China. Basic media were composed of 100 g sugar, 10 g yeast extract (Oxoid Corporation, Basingstoke, Hampshire, UK), 5 g CaCO_3_ and 1 liter distilled water, wherein the pH value of the media was adjusted to 5.0. The basic culture media were sterilized at 121 °C in an autoclave for 45 min, and then cooled to room temperature. The sugar solutions prepared from 12.7 wt % granular sugar content were sterilized and mixed with the basic media prepared above. Portions (ca.100 mL) of the sugar added media were poured into 250 mL Erlenmeyer flasks at prior to inoculation. The Acetobacter xylinum was then cultivated in the granular sugar added culture media prepared above at the optimum temperature at 30 °C, pH value at 5, sugar content at 12.7 wt % and an air flow rate of 1.25 m/s for 14 days. After metabolism, the bacterial cellulose products were washed and stirred in a beaker with distilled water for 40 min, and then repeatedly washed with fresh distilled water ten times to remove bacterial cells, residual sugars, salts and other metabolites. The purified bacterial cellulose products were then dried in an oven at 80 °C for 24 h before further characterization. As characterized in our previous investigation [47], typical reticulated rodlike feature with dimensions of 0.1–1 μm in length and 20–80 nm in diameter was observed for purified bacterial cellulose nanofiber products prepared in this study. The purified bacterial cellulose nanofiber products are with an extraordinary high specific surface area at 393.7 m^2^/g.

Before gelatinization, tapioca starches were modified using SHMP at 55 °C in a water bath for 3 h. In which, 50 g tapioca starch, 50 mL water and various contents of SHMP together with appropriate amounts of sodium carbonate were used to adjust the PH values of mixtures to 10.5 before modification. After reaction, the SHMP modified tapioca solutions were then filtered and washed with distilled water until neutral. Some of the reaction between SHMP and tapioca starch molecules is likely to crosslink tapioca starch molecules, but only to a very limited extent, because one can barely find the presence of insoluble gel of crosslinked tapioca starches during the filtration processes. Prior to gelatinization, 0.01 g BC nanofibers and 20 g glycerol were added and mixed with the SHMP modified tapioca solutions prepared above, in which the BC nanofibers and SHMP were used to improve the waterproof and strength retention properties of TPS, respectively. The above prepared mixtures were gelatinized in 250 mL flask at 90 °C under stirring condition for 15 min. The SHMP modified TPS and PLA resins were dried in an air dry oven and then in a vacuum dry oven both at 80 °C for 24 h to have a water content below 1 and 0.1 wt %, respectively. The dried SHMP modified TPS resins were then melt-blended with 25 wt % of PLA in a Changzhou Suyuan SU-70ML internal mixer at 180 °C for 3.5 min to improve their processibility, waterproof and strength retention properties. Table 1 summarized the sample codes and compositions of TPS, TPS_100_BC_0.02_, TPS_100_BC_0.02_SHMP_x_ and (TPS_100_BC_0.02_SHMP_x_)_75_PLA_25_ specimens prepared in this study.

**Table 1:** Sample codes and compositions of TPS, TPS_100_BC_0.02_, TPS_100_BC_0.02_SHMP_x_ and (TPS_100_BC_0.02_SHMP_x_)_75_PLA_25_ specimens.

Sample Codes	Starch Content (Parts)	BC Content (Parts)	SHMP Content (Parts)	PLA Content (Parts)
TPS	100	0	0	0
TPS_100_BC_0.02_	100	0.02	0	0
TPS_100_BC_0.02_SHMP_4_	100	0.02	4	0
TPS_100_BC_0.02_SHMP_8_	100	0.02	8	0
TPS_100_BC_0.02_SHMP_10_	100	0.02	10	0
TPS_100_BC_0.02_SHMP_16_	100	0.02	16	0
TPS_100_BC_0.02_SHMP_32_	100	0.02	32	0
(TPS_100_BC_0.02_)_75_PLA_25_	75	0.015	0	25
(TPS_100_BC_0.02_SHMP_4_)_75_PLA_25_	75	0.015	3.00	25
(TPS_100_BC_0.02_SHMP_8_)_75_PLA_25_	75	0.015	6.00	25
(TPS_100_BC_0.02_SHMP_10_)_75_PLA_25_	75	0.015	7.50	25
(TPS_100_BC_0.02_SHMP_16_)_75_PLA_25_	75	0.015	12.00	25
(TPS_100_BC_0.02_SHMP_32_)_75_PLA_25_	75	0.015	24.00	25

### 2.2. Fourier Transform Infrared Spectroscopy

Fourier transform infrared (FTIR) spectroscopic measurements of SHMP, TPS, TPS_100_BC_0.02_, TPS_100_BC_0.02_SHMP_x_ and (TPS_100_BC_0.02_SHMP_x_)_75_PLA_25_ specimens were recorded on a Nicolet Avatar 360 FTIR spectrophotometer at 25 °C, wherein 32 scans with a spectral resolution 1 cm^−1^ were collected during each spectroscopic measurement. Infrared spectra of SHMP, TPS, TPS_100_BC_0.02_, TPS_100_BC_0.02_SHMP_x_ and (TPS_100_BC_0.02_SHMP_x_)_75_PLA_25_ specimens were determined using the conventional KBr disk method. All the specimens were ground and mixed with KBr disk and then dried at 60 °C for 30 min. The film specimens used in this study were prepared sufficiently thin enough to obey the Beer-Lambert law.

### 2.3. Moisture Contents

Moisture contents of initial and conditioned TPS, TPS_100_BC_0.02_,TPS_100_BC_0.02_SHMP_x_ and (TPS_100_BC_0.02_GA_x_)_75_PLA_25_ specimens were determined using a Shanghai Jingke DHS16-A infrared moisture meter at temperatures ranging from 25 to 120 °C for 30 min.

### 2.4. Thermal Properties

Thermal properties of TPS, TPS_100_BC_0.02,_ TPS_100_BC_0.02_SHMP_x_ and (TPS_100_BC_0.02_SHMP_x_)_75_PLA_25_ were determined at 25 °C using a Du Pont 2010 differential scanning calorimetry (DSC). All scans were carried out at a heating rate of 20 °C/min and under flowing nitrogen at 25 mL/min. The instrument was calibrated using pure indium. Samples weighing about 0.5 mg were placed in standard aluminum sample pans for determination of their melting temperatures.

### 2.5. Wide Angle X-ray Diffraction Analyses

Wide angle X-ray diffraction (WAXD) patterns of TPS, TPS_100_BC_0.02_, TPS_100_BC_0.02_SHMP_x_ and (TPS_100_BC_0.02_SHMP_x_)_75_PLA_25_ specimens were determined at 25 °C using a Shimadu XRD-6000 diffractometer equipped with a Ni-filtered CuKα radiation operated at 40kV and 100mA. Each specimen with 2 mm thickness was maintained stationary and scanned in the reflection mode from 5 to 30° at a scanning rate of 5° min^−1^.

### 2.6. Tensile, Impact and Their Retention Properties

The injected specimens used to determine the tensile and tensile retention properties, initial and retention values of impact strengths of TPS, TPS_100_BC_0.02_, TPS_100_BC_0.02_SHMP_x_ and (TPS_100_BC_0.02_SHMP_x_)_75_PLA_25_ specimens were prepared according to ASTM D638 type IV with a specimen thickness of 0.254 cm using a Wuhan Reiming SZ-05 mini-injection machine at 180 °C and then cooled in the mold at 80 °C for 30 s. Before injection, TPS, TPS_100_BC_0.02_, TPS_100_BC_0.02_SHMP_x_ and (TPS_100_BC_0.02_SHMP_x_)_75_PLA_25_ resins were dried in an air dry oven and then in a vacuum dry oven both at 80 °C for 24 h to have a water content below 1 wt %. The injected specimens were then determined using a Hung-Ta HT-9112 tension testing machine at 25 °C and a crosshead speed of 50 mm/min. A 35 mm gauge length was used during each tensile experiment. The values of tensile and tensile retention properties were obtained based on the average results of at least five tensile specimens. The initial and retention values of impact strengths of the specimens prepared above were then determined using a Go-Tech GT-7045-HML digital impact strength testing machine at 25 °C and an impact speed of 3.5 m/s. The initial and retention values of impact strengths of TPS, TPS_100_BC_0.02_, TPS_100_BC_0.02_SHMP_x_ and (TPS_100_BC_0.02_SHMP_x_)_75_PLA_25_ specimens were obtained based on the average results of at least five impact specimens.

### 2.7. Morphology Analyses

The fractured tensile specimens used for morphological analyses were obtained by tensile testing the injected specimens using a Hung-Ta HT-9112 tension testing machine at 25 °C and a crosshead speed of 50 mm/min. The surfaces of fractured specimens were then observed using a Hitachi S-3000N scanning electron microscope (SEM). Prior to morphological analyses, the fracture surfaces of the tensile specimens were gold-coated at 20 mA and 15 kV for 10 s.

## 3. Results and Discussion

### 3.1. Fourier Transform Infrared Spectroscopy

**Figure 1:**
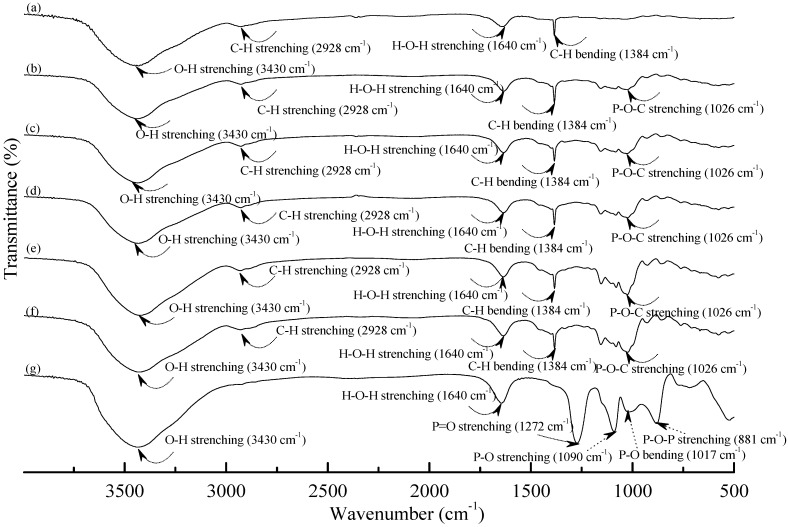
FTIR spectra of (**a**) TPS_100_BC_0.02_; (**b**) TPS_100_BC_0.02_SHMP_4_; (**c**) TPS_100_BC_0.02_SHMP_8_; (**d**) TPS_100_BC_0.02_SHMP_10_; (**e**) TPS_100_BC_0.02_SHMP_16_; (**f**) TPS_100_BC_0.02_SHMP_32_ and (**g**) SHMP specimens.

**Figure 2:**
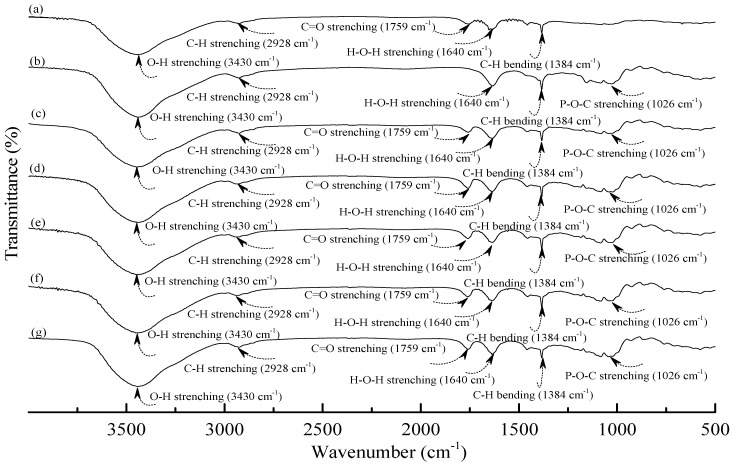
FTIR spectra of (**a**) PLA; (**b**) TPS_100_BC_0.02_SHMP_4_; (**c**) (TPS_100_BC_0.02_SHMP_4_)_75_PLA_25_; (**d**) (TPS_100_BC_0.02_SHMP_8_)_75_PLA_25_; (**e**) (TPS_100_BC_0.02_SHMP_10_)_75_PLA_25_; (**f**) (TPS_100_BC_0.02_SHMP_16_)_75_PLA_25_ and (**g**) (TPS_100_BC_0.02_SHMP_32_)_75_PLA_25_ specimens.

**Scheme 1:**
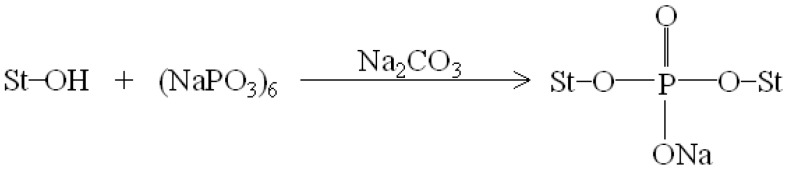
Reaction mechanism of sodium hexametaphosphate and starch molecules [47].

Figure 1 and Figure 2 illustrate typical Fourier transform infrared (FTIR) spectra of sodium hexametaphosphate (SHMP), TPS_100_BC_0.02_, TPS_100_BC_0.02_SHMP_x_ and (TPS_100_BC_0.02_SHMP_x_)_75_PLA_25_ specimens. Four distinctive absorption bands placed at 881, 1017, 1090 and 1272 cm^−1^ corresponding to the motions of P–O–P stretching, P–O bending, P–O and P=O stretching vibrations, respectively, were found on the FTIR spectrum of SHMP specimen [46] (see Figure 1g). Two other distinctive absorption bands placed at 1640 and 3430 cm^−1^ corresponding to motions of H–O–H and O–H stretching vibrations of absorbed water molecules were also found on FTIR spectrum of SHMP specimen [47]. As shown in Figure 1a, FTIR spectrum of the TPS_100_BC_0.02_ specimen exhibited four distinctive absorption bands placed at 1384, 1640, 2928 and 3430 cm^−1^, which were generally attributed to the motion of C–H bending, H–O–H, C–H and O–H stretching vibrations, respectively [47]. In addition to the C–H bending, H–O–H, C–H and gradually strengthened O–H stretching vibration bands, a new absorption band placed at 1026 cm^−1^ corresponding to ester (P–O–C) [48] stretching vibration gradually grew on FTIR spectra of TPS_100_BC_0.02_SHMP_x_ and (TPS_100_BC_0.02_SHMP_x_)_75_PLA_25_ series specimens as their SHMP contents increased (see Figure 1b–f and Figure 2b–g). However, after modification with varying amounts of SHMP, the absorption bands originally corresponding to the motions of P–O–P stretching, P–O bending, P–O and P=O stretching vibrations of phosphate group of SHMP disappeared nearly completely in FTIR spectra of TPS_100_BC_0.02_SHMP_x_ and (TPS_100_BC_0.02_SHMP_x_)_75_PLA_25_ series specimens. The newly developed ester stretching bands and disappeared P–O–P stretching, P–O bending, P–O and P=O stretching bands of TPS_100_BC_0.02_SHMP_x_ series specimens are most likely due to the reaction of the hydroxyl groups of TPS_100_BC_0.02_ specimens with the phosphate groups of SHMP molecules during their modification processes. It is highly likely that crosslinking reaction between starch and SHMP molecules can occur to some extent. The possible reaction mechanism between hydroxyl groups of TPS_100_BC_0.02_ specimens and phosphate groups of SHMP molecules is illustrated in Scheme 1 [47].

As shown in Figure 2a, PLA.specimen exhibited five distinctive absorption bands centered at 1384, 1640, 1759, 2928 and 3430 cm^−1^ corresponding to the motions of C–H bending vibration, H–O–H, C=O, C–H and O–H stretching vibrations bands [49,50,51], respectively. After blending 25 wt % PLA with TPS_100_BC_0.02_SHMP_x_, the FTIR spectra of (TPS_100_BC_0.02_SHMP_x_)_75_PLA_25_ series specimens look nearly the same as the integration of FTIR spectra of PLA and corresponding TPS_100_BC_0.02_SHMP_x_ specimens, in which no new vibration band but only vibration bands originally present in spectra of PLA and TPS_100_BC_0.02_SHMP_x_ specimens were found in FTIR spectra of (TPS_100_BC_0.02_SHMP_x_)_75_PLA_25_ series specimens, respectively. These results suggest that no distinctive chemical reaction or molecular interactions occurred during the melt-blending processes of PLA and TPS_100_BC_0.02_SHMP_x_ resins.

### 3.2. Morphology Analyses

**Figure 3:**
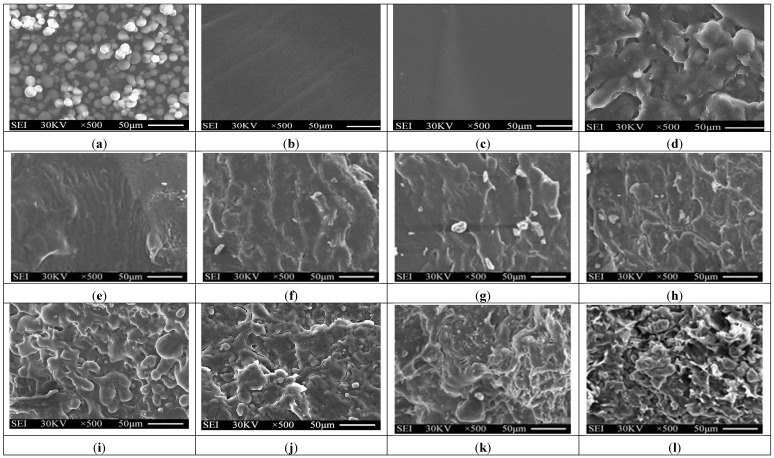
SEM micrographs of fracture surfaces of initial (**a**) Tapioca starch; (**b**) TPS; (**c**) TPS_100_BC_0.02_; (**d**) (TPS_100_BC_0.02_)_75_PLA_25_; (**e**) TPS_100_BC_0.02_SHMP_4_; (**f**) TPS_100_BC_0.02_SHMP_8_; (**g**) TPS_100_BC_0.02_SHMP_10_; (**h**) TPS_100_BC_0.02_SHMP_16_; (**i**) (TPS_100_BC_0.02_SHMP_4_)_75_PLA_25_; (**j**) (TPS_100_BC_0.02_SHMP_8_)_75_PLA_25_; (**k**) (TPS_100_BC_0.02_SHMP_10_)_75_PLA_25_ and (**l**) (TPS_100_BC_0.02_SHMP_16_)_75_PLA_25_ specimens.

Typical SEM micrographs of the fracture surfaces of tapioca starch, TPS, TPS_100_BC_0.02_SHMP_x_ and (TPS_100_BC_0.02_SHMP_x_)_75_PLA_25_ specimens are summarized in Figure 3. Granular tapioca starches with 5–10 μm in diameter were found on SEM micrograph of the original tapioca starches (see Figure 3a). The granular tapioca starches were completely dismantled and gelatinized as a continuous phase after gelatinization, in which only smooth characteristics were found on the fracture surface of TPS and TPS_100_BC_0.02_ specimens (see Figure 3b,c). After modification by SHMP, more ductile characteristics with drawn debris were found on the fracture surfaces of TPS_100_BC_0.02_SHMP_x_ and (TPS_100_BC_0.02_SHMP_x_)_75_PLA_25_ specimens (see Figure 3e–l). As evidenced by FTIR analyses in the previous section, this is most likely due to the crosslinking reaction of the hydroxyl groups of TPS_100_BC_0.02_ specimens with the phosphate groups of SHMP molecules. As shown in Figure 3d, clearly separated PLA droplets were found on (TPS_100_BC_0.02_)_75_PLA_25_ specimen that are attributed to the incompatibility between TPS_100_BC_0.02_ and PLA molecules during their melt-blending processes. In contrast, significantly less and smaller separated PLA droplets were found on fracture surfaces of (TPS_100_BC_0.02_SHMP_x_)_75_PLA_25_ specimens than were found for the (TPS_100_BC_0.02_)_75_PLA_25_ specimen. These results clearly suggested that the SHMP modified TPS_100_BC_0.02_SHMP_x_ molecules are much more compatible with PLA molecules.

### 3.3. Moisture Contents

**Figure 4:**
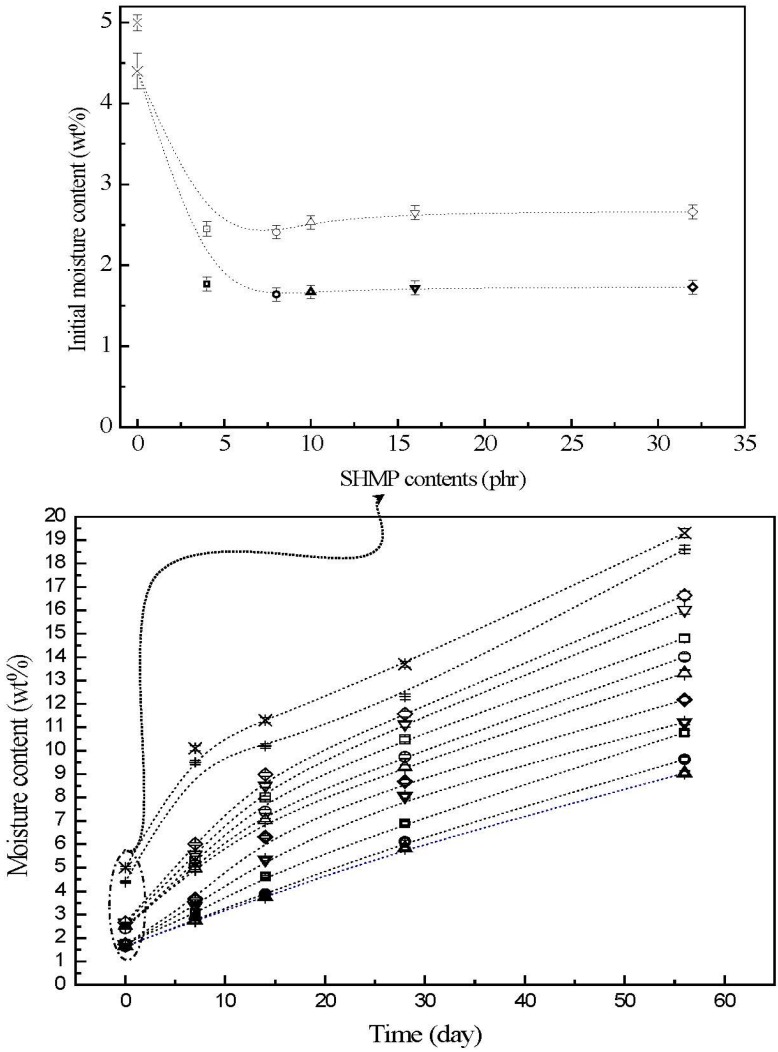
The moisture contents of initial and conditioned TPS (+), TPS_100_BC_0.02_ (▷), TPS_100_BC_0.02_SHMP_4_ (□), TPS_100_BC_0.02_ SHMP_8_ (○), TPS_100_BC_0.02_ SHMP_10_ (△), TPS_100_BC_0.02_SHMP_16_ (▽), TPS_100_BC_0.02_SHMP_32_ (◇), (TPS_100_BC_0.02_SHMP_4_)_75_PLA_25_ (**□**), (TPS_100_BC_0.02_SHMP_8_)_75_PLA_25_ (**○**), (TPS_100_BC_0.02_SHMP_10_)_75_PLA_25_ (**△**), (TPS_100_BC_0.02_SHMP_16_)_75_PLA_25_ (**▽**) and (TPS_100_BC_0.02_SHMP_32_)_75_PLA_25_ (**◇**) specimens. (Symbol (Ι) represents the error bar).

The moisture contents of initial and conditioned TPS, TPS_100_BC_0.02_, TPS_100_BC_0.02_SHMP_x_ and (TPS_100_BC_0.02_SHMP_x_)_75_PLA_25_ specimens are summarized in Figure 4. The initial TPS and TPS_100_BC_0.02_ specimens exhibited relatively high moisture contents at 5.0% and 4.4%, respectively. After remaining at 20 °C/50% RH for varying amounts of time, the moisture contents of conditioned TPS and TPS_100_BC_0.02_ specimens increased significantly from 5.0% and 4.4% to 10.1% and 9.5%, 13.7% and 12.3% and then to 19.6% and 19.3%, respectively, as the conditioning time increased from 0 to 7, 28 and to 56 days. After modification with varying amounts of SHMP, the moisture contents of initial TPS_100_BC_0.02_SHMP_x_ and (TPS_100_BC_0.02_SHMP_x_)_75_PLA_25_ specimens were reduced significantly to around 2.4% and 1.6%, respectively. The moisture contents of all conditioned TPS_100_BC_0.02_SHMP_x_ and (TPS_100_BC_0.02_SHMP_x_)_75_PLA_25_ specimens are significantly lower than those of corresponding conditioned TPS and TPS_100_BC_0.02_ specimens conditioned at 20 °C/50% RH for the same amounts of time, in which aged (TPS_100_BC_0.02_SHMP_x_)_75_PLA_25_ specimens exhibited even lower moisture contents than the corresponding conditioned TPS_100_BC_0.02_SHMP_x_ specimens without blending with 25 wt % of PLA. Moreover, it is noteworthy that conditioned (TPS_100_BC_0.02_SHMP_10_)_75_PLA_25_ specimens exhibited significantly lower moisture contents than conditioned (TPS_100_BC_0.02_SHMP_x_)_75_PLA_25_ specimens modified with SHMP contents other than 10 part per hundred parts of TPS resin (phr). In fact, after conditioning at 20 °C/50% relative humidity for 56 days, the moisture contents of conditioned (TPS_100_BC_0.02_SHMP_10_)_75_PLA_25_ specimens reached only 8.0%, which is less than half of the moisture contents of those of corresponding conditioned TPS and TPS_100_BC_0.02_ specimens.

As evidenced by FTIR analyses in the previous section, significant amounts of hydroxyl groups of starch molecules were reacted with phosphate groups of SHMP molecules into ester functional groups during the modification processes of TPS_100_BC_0.02_SHMP_x_ specimens. Apparently, the significant improvement in waterproof properties of the initial and conditioned TPS_100_BC_0.02_SHMP_x_ and/or (TPS_100_BC_0.02_SHMP_x_)_75_PLA_25_ specimens is mainly due to the efficient blocking of the moisture-absorbing hydroxyl groups of starch molecules present in TPS_100_BC_0.02_SHMP_x_ specimens during their modification processes. However, excess amounts of relatively large SHMP molecules can no longer react with the hydroxyl groups of starch molecules during the modification processes of TPS_100_BC_0.02_SHMP_x_ specimens. As a consequence, conditioned TPS_100_BC_0.02_SHMP_x_ specimens exhibited higher moisture contents than those of TPS_100_BC_0.02_SHMP_10_ specimen, since the remaining SHMP molecules are with strong hygroscopicity. In addition, blending TPS_100_BC_0.02_SHMP_x_ with inherently hydrophobic PLA can further prevent TPS_100_BC_0.02_SHMP_x_ from absorbing moisture and hence improve the waterproof properties of the initial and conditioned (TPS_100_BC_0.02_SHMP_x_)_75_PLA_25_ specimens.

### 3.4. Thermal Properties

**Figure 5:**
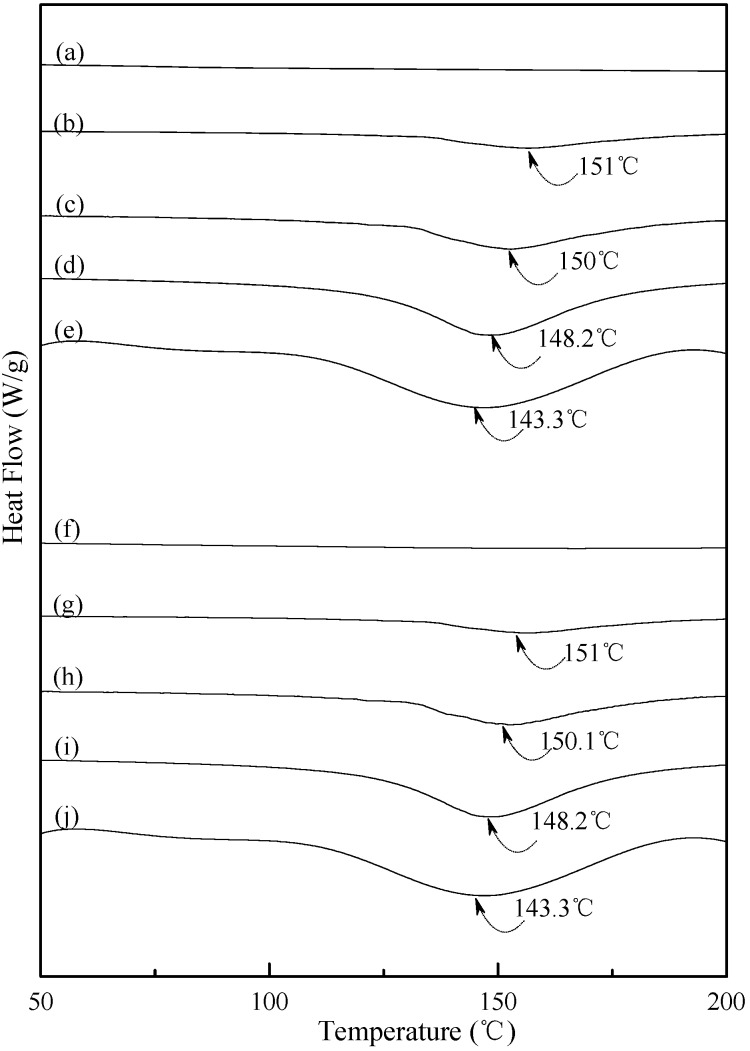
DSC thermograms of TPS specimens conditioned at 20 °C/50% RH for (**a**) 0; (**b**) 7; (**c**)14; (**d**) 28 and (**e**) 56 days, respectively; and TPS_100_BC_0.02_ specimens conditioned at 20 °C/50% RH for (**f**) 0; (**g**) 7; (**h**) 14; (**i**) 28 and (**j**) 56 days, respectively.

**Figure 6:**
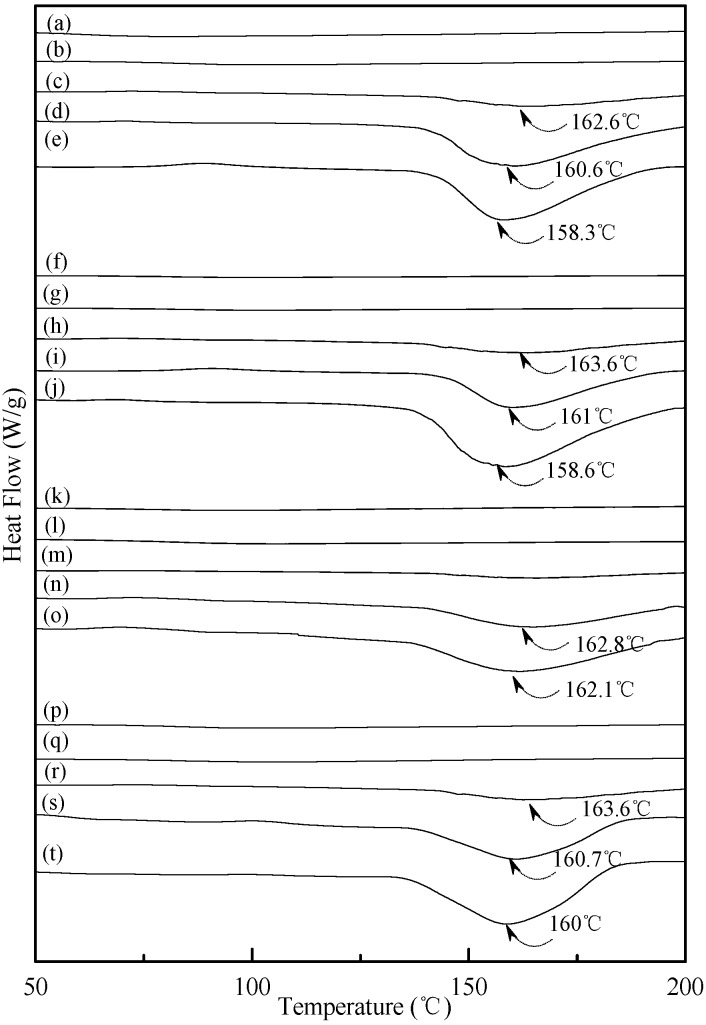
DSC thermograms of TPS_100_BC_0.02_SHMP_4_ specimens conditioned at 20 °C/50% RH for (**a**) 0; (**b**) 7; (**c**) 14; (**d**) 28 and (**e**) 56 days respectively; TPS_100_BC_0.02_SHMP_8_ specimens conditioned at 20 °C/50% RH for (**f**) 0; (**g**) 7; (**h**) 14; (**i**) 28 and (**j**)56 days, respectively; TPS_100_BC_0.02_SHMP_10_ specimens conditioned at 20 °C/50% RH for (**k**) 0; (**l**) 7; (**m**) 14; (**n**) 28 and (**o**) 56 days, respectively; and TPS_100_BC_0.02_SHMP_16_ specimens conditioned at 20 °C/50% RH for (**p**) 0; (**q**) 7; (**r**) 14; (**s**) 28 and (**t**) 56 days, respectively.

Typical DSC thermograms of TPS, TPS_100_BC_0.02_ and TPS_100_BC_0.02_SHMP_x_ specimens are shown in Figure 5 and Figure 6. Smooth thermograms without any endotherms were found for initial TPS, TPS_100_BC_0.02_ and TPS_100_BC_0.02_SHMP_x_ specimens (see Figure 5a,f, and Figure 6a,f,k,p). A new melting endotherm with a peak temperature at about 150 °C gradually appeared on the DSC thermograms of TPS and TPS_100_BC_0.02_ specimens, respectively, after they were conditioned at 20 °C/50% RH for 7 days or more than 7 days. The size of the new melting endotherm grew significantly as the conditioning time increased. However, as shown in Figure 5b–e and Figure 5g–j, the peak melting temperatures of conditioned TPS and TPS_100_BC_0.02_ specimens shifted from around 151.0 °C to 150.0 °C, 148.2 °C and then to 143.3 °C as the conditioning time increased from 7, 14 to 28 and 56 days, respectively. In contrast, one can barely find any endotherm on DSC thermograms of TPS_100_BC_0.02_SHMP_x_ specimens after they were conditioned at 20 °C/50% RH for less than 14 days (see Figure 6b,g,l,m,q). In fact, the thermograms of TPS_100_BC_0.02_SHMP_10_ specimen remained relatively smooth without any distinguished endotherm even after conditioning at 20 °C/50% RH for less than 28 days (see Figure 6l,m). Similarly, the peak melting temperatures of conditioned TPS_100_BC_0.02_SHMP_4_ and TPS_100_BC_0.02_SHMP_16_ specimens were reduced from 162.6 °C and 163.6 °C to 158.3 °C and 160 °C, respectively, as the conditioning time values increased from 14 to 56 days (see Figure 6c–e and Figure 6r–t). The above results revealed that recrystallization of tapioca starch molecules of TPS, TPS_100_BC_0.02_ and/or TPS_100_BC_0.02_SHMP_x_ specimens only occurred after the specimens absorbed enough amounts of plasticizers (e.g., water) during their conditioning processes. Apparently, higher amounts of water molecules absorbed during longer conditioning processes of TPS, TPS_100_BC_0.02_ and/or TPS_100_BC_0.02_SHMP_x_ specimens facilitate the crystallization of the tapioca starches into higher amounts of tapioca starch crystals but with lower melting temperatures, respectively.

**Figure 7:**
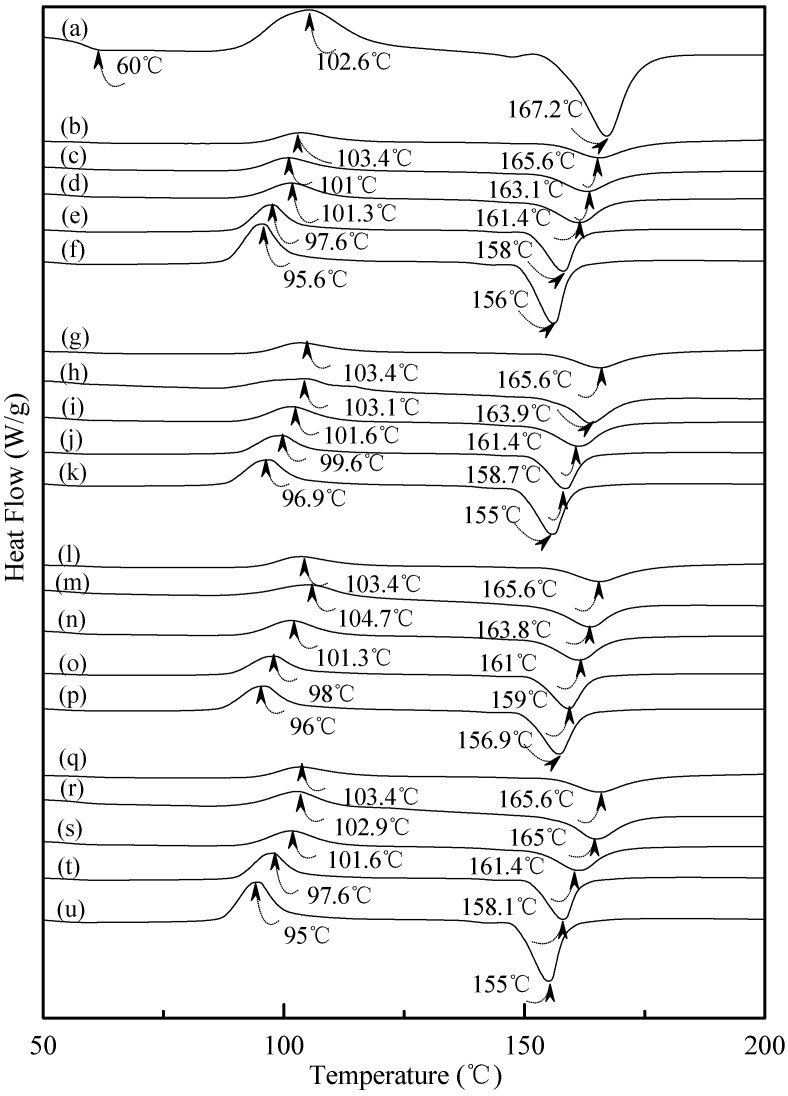
DSC thermograms of (**a**) PLA, (TPS_100_BC_0.02_SHMP_4_)_75_PLA_25_ specimens conditioned at 20 °C/50% RH for (**b**) 0; (**c**) 7; (**d**) 14; (**e**) 28 and (**f**) 56 days respectively; (TPS_100_BC_0.02_SHMP_8_)_75_PLA_25_ specimens conditioned at 20 °C/50% RH for (**g**) 0; (**h**) 7; (**i**) 14; (**j**) 28 and (k)56 days, respectively; (TPS_100_BC_0.02_SHMP_10_)_75_PLA_25_ specimens conditioned at 20 °C/50% RH for (**l**) 0; (**m**) 7; (**n**) 14; (**o**) 28 and (**p**) 56 days, respectively; and (TPS_100_BC_0.02_SHMP_16_)_75_PLA_25_ specimens conditioned at 20 °C/50% RH for (**q**) 0; (**r**) 7; (**s**) 14; (**t**) 28 and (**u**) 56 days, respectively.

Figure 7 exhibited typical DSC thermograms of (TPS_100_BC_0.02_SHMP_x_)_75_PLA_25_ specimens. As shown in Figure 7a, a distinguished melting endotherm with a peak melting temperature 167.2 °C was found on the DSC thermogram of PLA specimen. Moreover, a glass transition at 60.0 °C and a recrystallization exotherm with a peak temperature at 102.6 °C was found on the DSC thermogram of PLA specimen. After blending 25 wt % PLA with TPS_100_BC_0.02_SHMP_x_, the DSC thermograms of (TPS_100_BC_0.02_SHMP_x_)_75_PLA_25_ series specimens look nearly the same as the integration of thermograms of PLA and corresponding TPS_100_BC_0.02_SHMP_x_ specimens, respectively. It is interesting to note that thermograms of conditioned (TPS_100_BC_0.02_SHMP_x_)_75_PLA_25_ specimens remained relatively unchanged regardless of their conditioning time at 20 °C/50% RH. In contrast to those TPS_100_BC_0.02_SHMP_x_ specimens conditioned at 20 °C/50% RH for 28 or 56 days, one can barely find the newly developed melting endotherm on thermograms of (TPS_100_BC_0.02_SHMP_x_)_75_PLA_25_ specimens, even when they were conditioned at 20 °C/50% RH for 56 days.

### 3.5. Wide Angle X-ray Diffraction

**Figure 8:**
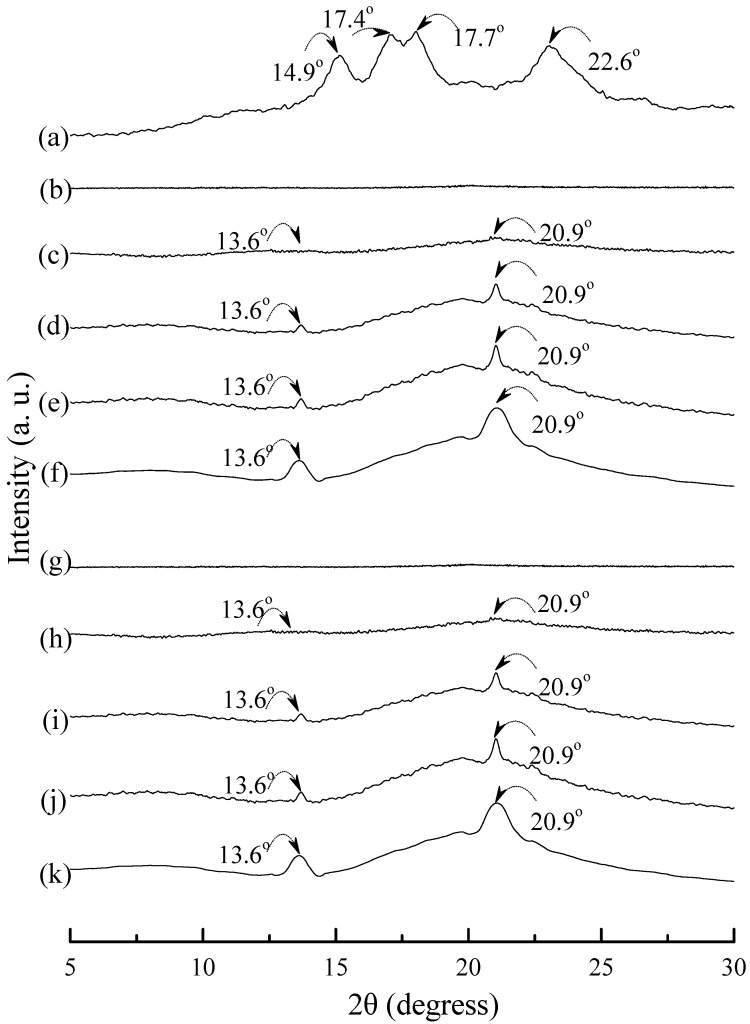
Wide-angle X-ray diffraction patterns of (**a**) tapioca, TPS specimens conditioned at 20 °C/50% RH for (**b**) 0; (**c**) 7; (**d**) 14; (**e**) 28 and (**f**) 56 days, respectively; and TPS_100_BC_0.02_ specimens conditioned at 20 °C/50% RH for (**g**) 0; (**h**) 7; (**i**) 14; (**j**) 28 and (**k**) 56 days, respectively.

**Figure 9:**
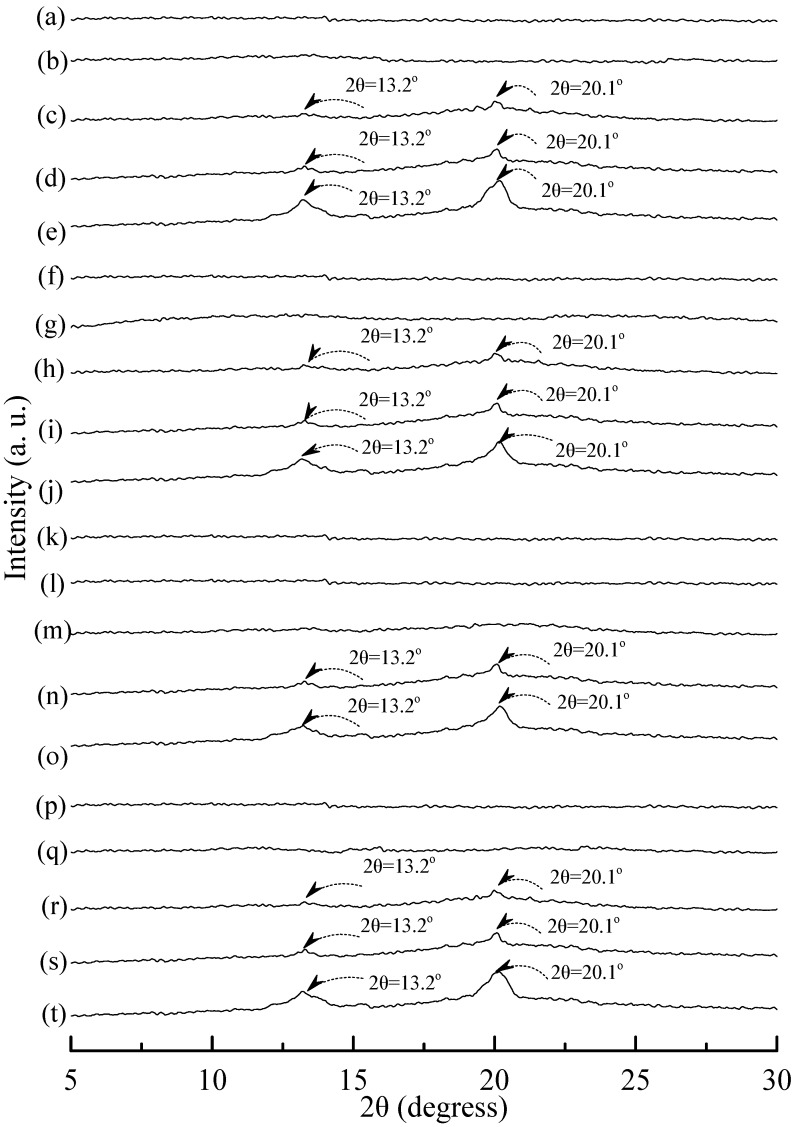
Wide-angle X-ray diffraction patterns of TPS_100_BC_0.02_SHMP_4_ specimens conditioned at 20 °C/50% RH for (**a**) 0; (**b**) 7; (**c**) 14; (**d**) 28 and (**e**) 56 days respectively; TPS_100_BC_0.02_SHMP_8_ specimens conditioned at 20 °C/50% RH for (**f**) 0; (**g**) 7; (**h**) 14; (**i**) 28 and (**j**)56 days, respectively; TPS_100_BC_0.02_SHMP_10_ specimens conditioned at 20 °C/50% RH for (**k**) 0; (**l**) 7; (**m**) 14; (**n**) 28 and (**o**) 56 days, respectively; and TPS_100_BC_0.02_SHMP_16_ specimens conditioned at 20 °C/50% RH for (**p**) 0; (**q**) 7; (**r**) 14; (**s**) 28 and (**t**) 56 days, respectively.

**Figure 10:**
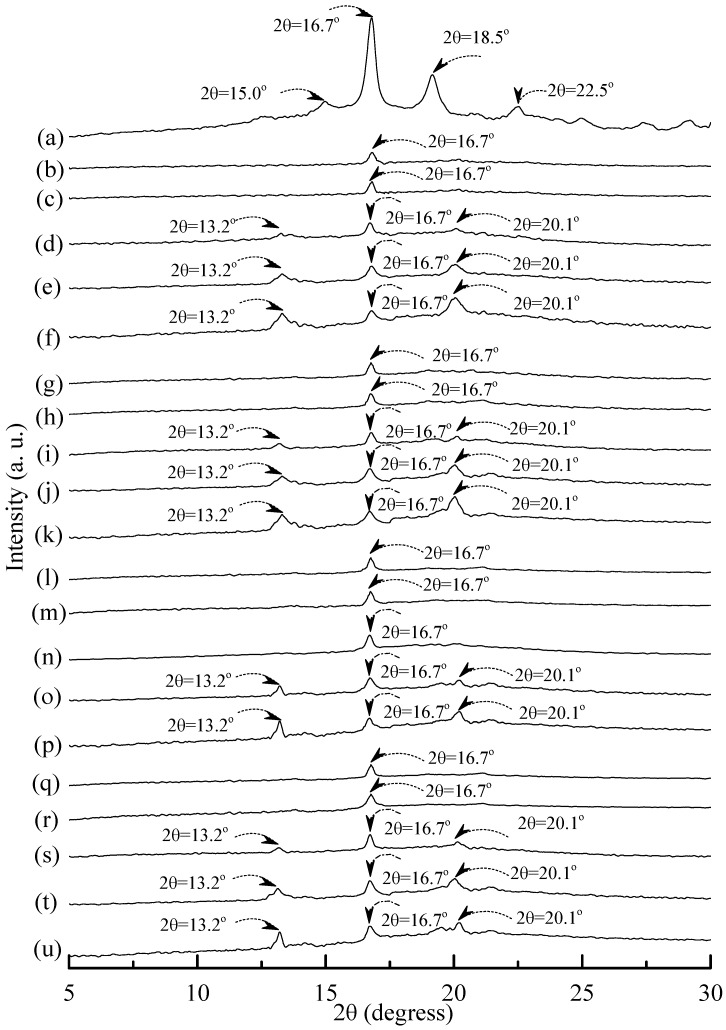
Wide-angle X-ray diffraction patterns of (**a**) PLA, (TPS_100_BC_0.02_SHMP_4_)_75_PLA_25_ specimens conditioned at 20 °C/50% RH for (**b**) 0; (**c**) 7; (**d**) 14; (**e**) 28 and (**f**) 56 days respectively; (TPS_100_BC_0.02_SHMP_8_)_75_PLA_25_ specimens conditioned at 20 °C/50% RH for (**g**) 0; (**h**) 7; (**i**) 14; (**j**) 28 and (**k**)56 days, respectively; (TPS_100_BC_0.02_SHMP_10_)_75_PLA_25_ specimens conditioned at 20 °C/50% RH for (**l**) 0; (**m**) 7; (**n**) 14; (**o**) 28 and (**p**) 56 days, respectively; and (TPS_100_BC_0.02_SHMP_16_)_75_PLA_25_ specimens conditioned at 20 °C/50% RH for (**q**) 0; (**r**) 7; (**s**) 14; (**t**) 28 and (**u**) 56 days, respectively.

Typical wide angle X-ray diffraction (WAXD) patterns of tapioca, initial and conditioned TPS, TPS_100_BC_0.02_, TPS_100_BC_0.02_SHMP_x_, (TPS_100_BC_0.02_SHMP_x_)_75_PLA_25_ and PLA specimens are shown in Figure 8, Figure 9 and Figure 10. As shown in Figure 8a, distinguished diffraction peaks centered at 14.9°, 17.4°, 17.7° and 22.6° were found on WAXD diffraction patterns of tapioca starches. These diffraction peaks most likely correspond to A-type starch crystals with strong reflections at 2θ around 14.8° and an unresolved doublet at around 17° and 22.6° [37,52,53]. After gelatinization, the diffraction peaks corresponding to A-type starch crystals disappeared near completely on the WAXD diffraction pattern of the initial TPS, and TPS_100_BC_0.02_ specimens (see Figure 8b–g). Two new diffraction peaks centered at 2θ = 13.6° and 20.9° appeared gradually on WAXD patterns of TPS and TPS_100_BC_0.02_ specimens, respectively, after they were conditioned at 20 °C/50% RH for 7 days or more than 7 days. In fact, the sizes of two new diffraction peaks grew significantly, as the conditioning time increased from 0 to 56 days (see Figure 8b–f and Figure 8g–k). The two new diffraction peaks were reported to originate from diffraction of V_H_-type crystallinity [29], which was induced during their plasticization processes. In contrast, one can barely find the two new diffraction peaks on WAXD patterns of TPS_100_BC_0.02_SHMP_x_ specimens conditioned at 20 °C/50% RH for less than 14 days (see Figure 9). The two new diffraction peaks of most of TPS_100_BC_0.02_SHMP**_x_** specimens reappeared and grew gradually, as the conditioning time were equal to or more than 14 days (see Figure 9c–e, h–j, n–o and r–t). In which, WAXD patterns of TPS_100_BC_0.02_SHMP_10_ specimen remained relatively smooth without any diffraction peak even after conditioning at 20 °C/50% RH for less than 28 days (see Figure 9k–m).

Distinguishable diffraction peaks centered at 2θ = 15°, 16.7°, 18.5° and 22.5° were found on the WAXD pattern of the PLA specimen (see Figure 10a). These diffraction peaks were reported to originate from the diffraction of α form PLA crystals [54]. After blending 25 wt % PLA with TPS_100_BC_0.02_SHMP_x_, one can only find a weak diffraction peak centered at 16.7° on WAXD diffraction patterns of (TPS_100_BC_0.02_SHMP_x_)_75_PLA_25_ specimens (see Figure 10). No additional diffraction peak was found on WAXD patterns of (TPS_100_BC_0.02_SHMP_x_)_75_PLA_25_ specimens, when they were conditioned at 20 °C/50% RH for less than 14 days. Two new diffraction peaks centered at 2θ = 13.2° and 20.1° gradually appeared on WAXD patterns of (TPS_100_BC_0.02_SHMP_x_)_75_PLA_25_ specimens conditioned at 20 °C/50% RH for 14 days or more than 14 days.

WAXD analyses revealed that A-type starch crystals originally present in granular tapioca starches were completely dismantled during their gelatinization processes. The new melting endotherm and diffraction peaks of V_H_-type crystals found in DSC thermograms and WAXD patterns of conditioned TPS or TPS_100_BC_0.02_ specimens, respectively, was attributed to the significant retrogradation of tapioca starch molecules occurred during their conditioning processes. During retrogradation, recrystallization of tapioca starch molecules of TPS and/or TPS_100_BC_0.02_ specimens occurred significantly in moisture rich environment, since TPS or TPS_100_BC_0.02_ specimens can easily absorb moisture during their conditioning processes. However, one can barely find any new melting endotherm or diffraction peaks on DSC thermograms or WAXD patterns of TPS_100_BC_0.02_SHMP_x_ and/or (TPS_100_BC_0.02_SHMP_x_)_75_PLA_25_ specimens, respectively, even after they were conditioned at 20 °C/50% RH for less than 28 days. Apparently, this is due to the significant improvement in waterproof properties of the TPS_100_BC_0.02_SHMP_x_ and/or (TPS_100_BC_0.02_SHMP_x_)_75_PLA_25_ specimens, since the moisture-absorbing hydroxyl groups of starch molecules were successfully reacted with the phosphate groups of SHMP molecules during the modification processes of TPS_100_BC_0.02_SHMP_x_ specimens.

### 3.6. Tensile and Tensile Retention Properties

**Figure 11:**
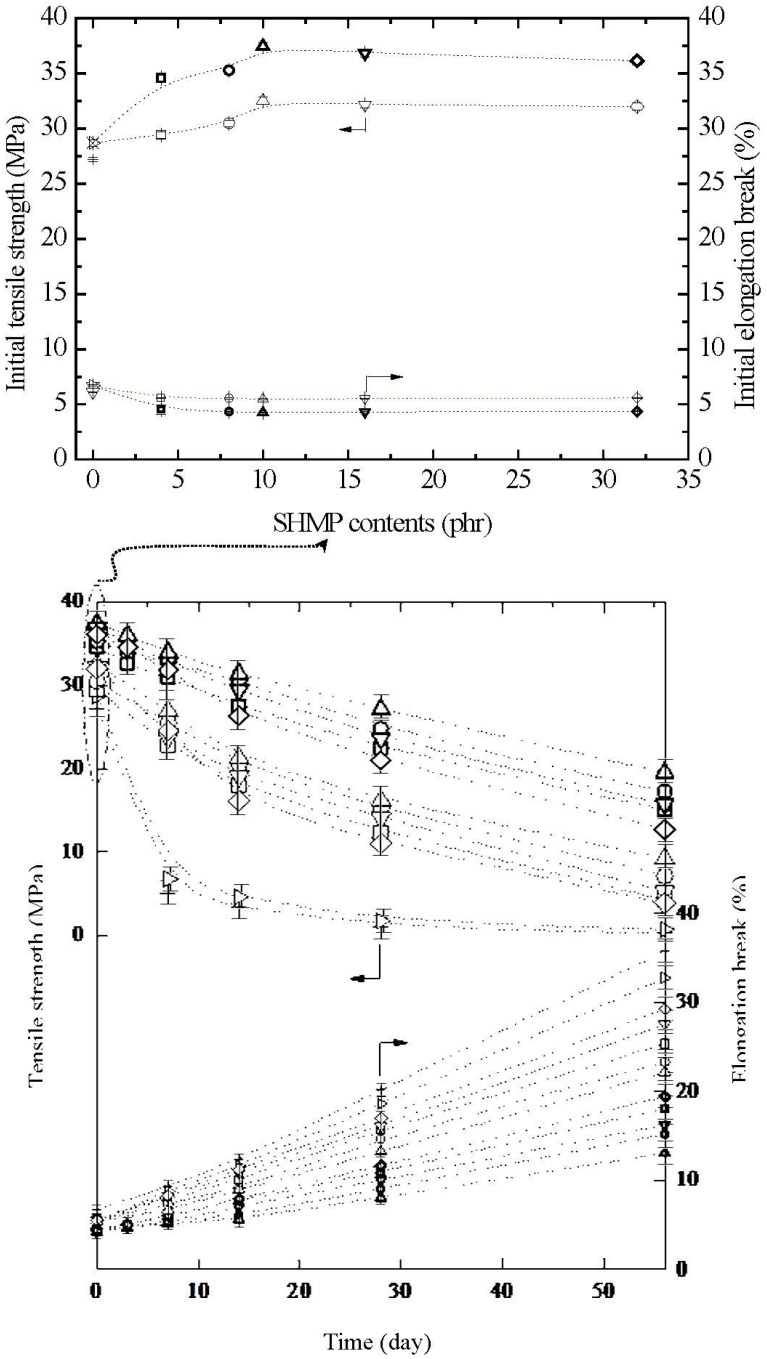
Tensile strength and elongation at break of initial and conditioned TPS (+,+), TPS_100_BC_0.02_ (▷,▷), TPS_100_BC_0.02_SHMP_4_ (□,□), TPS_100_BC_0.02_SHMP_8_ (○,○), TPS_100_BC_0.02_ SHMP_10_ (△,△), TPS_100_BC_0.02_SHMP_16_ (▽,▽), TPS_100_BC_0.02_SHMP_32_ (◇,◇), (TPS_100_BC_0.02_SHMP_4_)_75_PLA_25_ (**□**,**□**), (TPS_100_BC_0.02_SHMP_8_)_75_PLA_25_ (**○**,**○**), (TPS_100_BC_0.02_SHMP_10_)_75_PLA_25_ (**△**,**△**), (TPS_100_BC_0.02_SHMP_16_)_75_PLA_25_ (**▽**,**▽**) and (TPS_100_BC_0.02_SHMP_32_)_75_PLA_25_ (**◇**,**◇**) specimens conditioned at 20 °C/50% RH for varying amounts of time. (Symbol (Ι) represents the error bar).

The initial and retention values of tensile strength (σ_f_) and elongation at break (ɛ_f_) of TPS, TPS_100_BC_0.02_, TPS_100_BC_0.02_SHMP_x_ and (TPS_100_BC_0.02_SHMP_x_)_75_PLA_25_ specimens are summarized in Figure 11. Relatively high σ_f_ and ɛ_f_ values at 27.2 MPa/6.7% and 28.7 MPa/6.1% were found for the initial TPS and TPS_100_BC_0.02_ specimens, respectively. However, after maintaining the specimens at 20 °C/50% RH for certain amounts of time, the σ_f_ retention values of TPS and TPS_100_BC_0.02_ specimens were reduced rapidly from 27.2 MPa/28.7 MPa to 5.1 MPa/6.8 MPa to 1.3 MPa/1.8 MPa and then to 0.3 MPa/0.6 MPa, respectively, as the conditioning time increased from 0 to 7, 28 and to 56 days. In contrast, the ɛ_f_ retention values of of TPS and TPS_100_BC_0.02_ specimens increased significantly from 6.7%/6.1% to 9.3%/8.8%, 20.2%/18.6% and then to 35.8%/32.8%, respectively, as the conditioning time increased from 0 to 7, 28 and to 56 days. Apparently, initial and conditioned TPS_100_BC_0.02_ specimen with very small amounts of BC nanofibers exhibited significantly higher σ_f_ values but lower ɛ_f_ values than those of corresponding TPS specimens conditioned at 20 °C/50% RH for the same amounts of time.

After modification with varying amounts of SHMP during TPS_100_BC_0.02_ gelatinization processes, the σ_f_ and ɛ_f_ values of initial TPS_100_BC_0.02_SHMP_x_ and (TPS_100_BC_0.02_SHMP_x_)_75_PLA_25_ specimens increased and reduced significantly to a maximal and minimal value at 32.5 MPa/37.4 MPa and 5.5%/4.3%, respectively, as their SHMP contents reached an optimal value at 10 phr. However, after conditioning at 20 °C/50% RH for varying amounts of time, the σ_f_ retention values of TPS_100_BC_0.02_SHMP_x_ and (TPS_100_BC_0.02_SHMP_x_)_75_PLA_25_ specimens were significantly higher than those of TPS and TPS_100_BC_0.02_ specimens conditioned for the same amounts of time, whereas significantly lower ɛ_f_ retention values were found for conditioned TPS_100_BC_0.02_SHMP_x_ and (TPS_100_BC_0.02_SHMP_x_)_75_PLA_25_ specimens than those of TPS and TPS_100_BC_0.02_ specimens conditioned for the same amounts of time. Conditioned (TPS_100_BC_0.02_SHMP_x_)_75_PLA_25_ specimens exhibited significantly higher σ_f_ but lower ɛ_f_ retention values than corresponding TPS_100_BC_0.02_SHMP_x_ specimens conditioned for the same amounts of time but without blending with 25 wt % of PLA (see Figure 11). Moreover, it is noteworthy that conditioned TPS_100_BC_0.02_SHMP_10_ or (TPS_100_BC_0.02_SHMP_10_)_75_PLA_25_ specimens exhibited the highest σ_f_ but the lowest ɛ_f_ retention values than other TPS_100_BC_0.02_SHMP_x_ or (TPS_100_BC_0.02_SHMP_x_)_75_PLA_25_ specimens conditioned for the same amounts of time but modified with SHMP contents other than 10 phr, respectively. In fact, after conditioning at 20 °C/50% RH for 56 days, the σ_f_ and ɛ_f_ retention values of (TPS_100_BC_0.02_SHMP_10_)_75_PLA_25_ specimen remained at 19.5 MPa and 13.1%, respectively, which is equivalent to 52% and 3.1 times its initial σ_f_ and ɛ_f_ value, respectively, and is 32.5 times and 40% those of TPS_100_BC_0.02_ specimen conditioned for 56 days, respectively.

### 3.7. Initial and Retention Values of Impact Strengths

**Figure 12:**
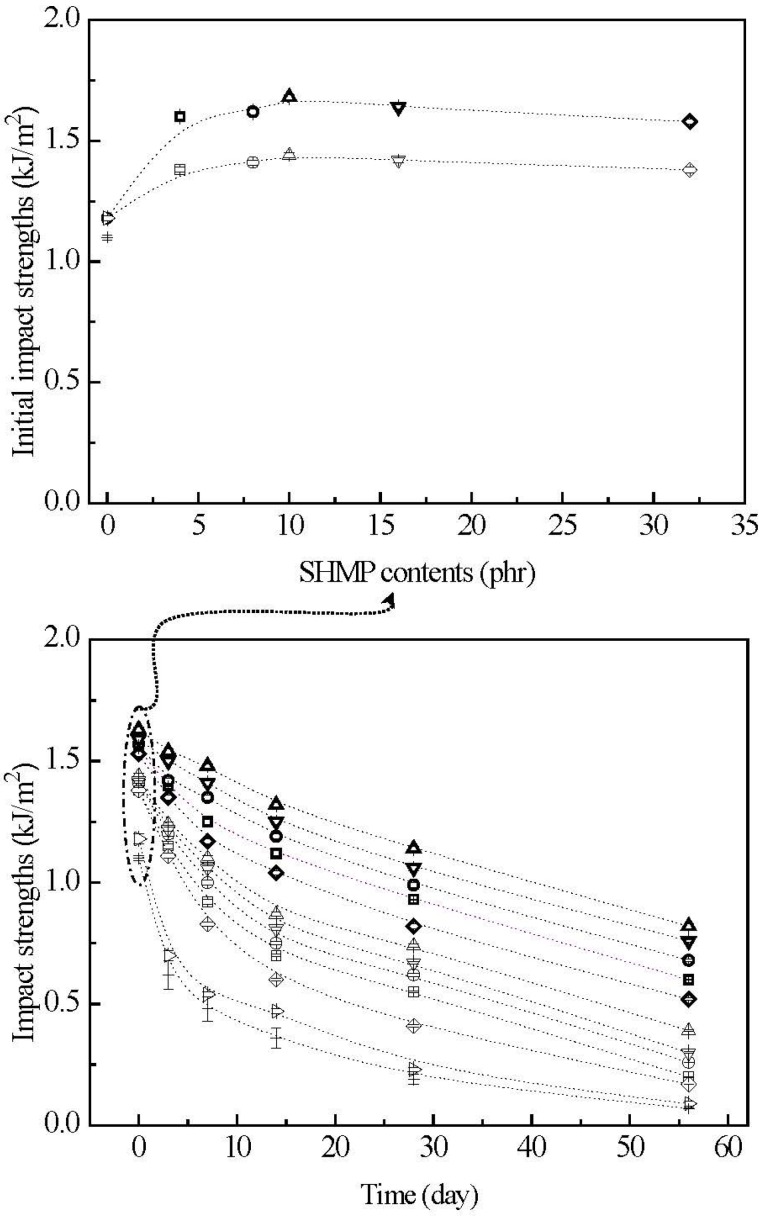
The impact strengths of initial and conditioned TPS (+), TPS_100_BC_0.02_ (▷), TPS_100_BC_0.02_SHMP_4_ (□), TPS_100_BC_0.02_ SHMP_8_ (○), TPS_100_BC_0.02_ SHMP_10_ (△), TPS_100_BC_0.02_SHMP_16_ (▽), TPS_100_BC_0.02_SHMP_32_ (◇), (TPS_100_BC_0.02_SHMP_4_)_75_PLA_25_ (□), (TPS_100_BC_0.02_SHMP_8_)_75_PLA_25_ (○), (TPS_100_BC_0.02_SHMP_10_)_75_PLA_25_ (△), (TPS_100_BC_0.02_SHMP_16_)_75_PLA_25_ (▽) and (TPS_100_BC_0.02_SHMP_32_)_75_PLA_25_ (◇) specimens conditioned at 20 °C/50% RH for varying amounts of time. (Symbol (Ι) represents the error bar).

The initial and retention values of impact strengths (I_s_) of TPS, TPS_100_BC_0.02_, TPS_100_BC_0.02_SHMP_x_ and (TPS_100_BC_0.02_SHMP_x_)_75_PLA_25_ specimens are summarized in Figure 12. Before conditioning at 20 °C/50% RH, TPS and TPS_100_BC_0.02_ specimens exhibited relatively low initial I_s_ values at 1.1 KJ/m^2^ and 1.2 KJ/m^2^, respectively. After maintaining at 20 °C/50% RH for certain amounts of time, the I_s_ values of conditioned TPS and TPS_100_BC_0.02_ specimens reduced rapidly from 1.1 kJ/m^2^/1.2 kJ/m^2^ to 0.4 kJ/m^2^/0.5 kJ/m^2^, 0.1 kJ/m^2^/0.2 kJ/m^2^ and then to 0.07 kJ/m^2^/0.09 kJ/m^2^, respectively, as the conditioning time increased from 0 to 7, 28 and 56 days. Apparently, initial and TPS_100_BC_0.02_ specimens with very small amounts of BC nanofibers exhibited significantly higher I_s_ values than those of corresponding TPS specimens conditioned at 20 °C/50% RH for the same amounts of time.

After modification with varying amounts of SHMP during gelatinization processes of TPS_100_BC_0.02_, the initial I_s_ values of TPS_100_BC_0.02_SHMP_x_ and (TPS_100_BC_0.02_SHMP_x_)_75_PLA_25_ specimens increased to a maximal value, as their SHMP contents reached an optimal value at 10 phr. However, after conditioning at 20 °C/50% RH for certain amounts of time, the I_s_ retention values of all TPS_100_BC_0.02_SHMP_x_ and (TPS_100_BC_0.02_SHMP_x_)_75_PLA_25_ specimens are significantly higher than those of corresponding conditioned TPS and TPS_100_BC_0.02_ specimens. Conditioned (TPS_100_BC_0.02_SHMP_x_)_75_PLA_25_ specimens exhibited significantly higher I_s_ retention values than corresponding TPS_100_BC_0.02_SHMP_x_ specimens conditioned for the same amounts of time but without blending with 25 wt % of PLA. Moreover, it is noteworthy that conditioned TPS_100_BC_0.02_SHMP_10_ or (TPS_100_BC_0.02_SHMP_10_)_75_PLA_25_ specimens showed significantly higher I_s_ retention values than other corresponding conditioned TPS_100_BC_0.02_SHMP_x_ or (TPS_100_BC_0.02_SHMP_x_)_75_PLA_25_ specimens modified with SHMP contents other than 10 phr, respectively. For instance, after conditioning at 20 °C/50% relative humidity for 56 days, the I_s_ retention value of (TPS_100_BC_0.02_SHMP_10_)_75_PLA_25_ specimen remained at 0.8 KJ/m^2^, which is equivalent to about 50% of its initial I_s_ value and 8.9 times those of TPS_100_BC_0.02_ specimen conditioned for the same amounts of time.

The rapid reduction in σ_f_ and I_s_ retention values but increase in ɛ_f_ retention values of the conditioned TPS and TPS_100_BC_0.02_ specimens is apparently due to the excessive amounts of moisture absorbed during their conditioning processes, because the absorbed water molecules can effectively plasticize, soften and recrystallize starch molecules during their conditioning processes. As a consequence, ɛ_f_ values of TPS and TPS_100_BC_0.02_ specimens increased significantly as the conditioning time increased, while their σ_f_ and I_s_ values reduced rapidly with the increase in conditioning time. In contrast, as evidenced by moisture content analyses in the previous section, the waterproof properties of TPS_100_BC_0.02_SHMP_x_ and (TPS_100_BC_0.02_SHMP_x_)_75_PLA_25_ specimens were significantly improved, because the moisture-absorbing hydroxyl groups of starch molecules were successfully blocked by reacting with proper amounts of phosphate groups of SHMP molecules during their modification processes. However, excess amounts of relatively large SHMP molecules can no longer react with the hydroxyl groups of starch molecules during the modification processes of TPS_100_BC_0.02_SHMP_x_ specimens. As a consequence, conditioned TPS_100_BC_0.02_SHMP_x_ specimens exhibited higher moisture contents than those of TPS_100_BC_0.02_SHMP_10_ specimen, since the remained SHMP molecules are with strong hygroscopicity. It is, therefore, reasonable to infer that TPS_100_BC_0.02_SHMP_x_ and (TPS_100_BC_0.02_SHMP_x_)_75_PLA_25_ specimens can exhibit significantly improved σ_f_ and I_s_ retention values but reduced ɛ_f_ retention values than those of conditioned TPS and/or TPS_100_BC_0.02_ specimens. Conditioned TPS_100_BC_0.02_SHMP_10_ and (TPS_100_BC_0.02_SHMP_10_)_75_PLA_25_ specimens exhibit the best σ_f,_ ɛ_f_ and I_s_ retention values compared to those of other corresponding conditioned TPS_100_BC_0.02_SHMP_x_ and (TPS_100_BC_0.02_SHMP_x_)_75_PLA_25_ specimens modified with SHMP contents other than 10 phr, respectively. In addition, the inherently hydrophobic PLA can further prevent TPS_100_BC_0.02_SHMP_x_ from absorbing moisture and hence, improve the waterproof properties of the initial and conditioned (TPS_100_BC_0.02_SHMP_x_)_75_PLA_25_ specimens. As a consequence, the conditioned (TPS_100_BC_0.02_SHMP_x_)_75_PLA_25_ specimens exhibited significantly higher σ_f_ and I_s_ retention but lower ɛ_f_ retention values than corresponding TPS_100_BC_0.02_SHMP_x_ specimens conditioned for the same amounts of time but without blending with 25 wt % of PLA.

## 4. Conclusions

Waterproof and strength retention properties of BC-reinforced TPS resins were successfully improved by reacting with SHMP molecules during their gelatinization processes. As evidenced by FTIR analyses, hydroxyl groups of TPS_100_BC_0.02_ resins were successfully reacted with the phosphate groups of SHMP molecules during their modification processes. The moisture contents of all conditioned TPS_100_BC_0.02_SHMP_x_ and (TPS_100_BC_0.02_SHMP_x_)_75_PLA_25_ specimens were significantly lower than those of corresponding conditioned TPS and TPS_100_BC_0.02_ specimens maintained at 20 °C/50% RH for the same amounts of time. In fact, for the same conditioning time, the moisture content values of initial and conditioned TPS_100_BC_0.02_SHMP_x_ and (TPS_100_BC_0.02_SHMP_x_)_75_PLA_25_ specimens were reduced to a minimal value, as their SHMP contents approached an optimal value at 10 phr. Apparently, the significant improvement in waterproof properties of the initial and conditioned TPS_100_BC_0.02_SHMP_x_ and/or (TPS_100_BC_0.02_SHMP_x_)_75_PLA_25_ specimens is mainly due to the efficient blocking of the moisture-absorbing hydroxyl groups of starch molecules during their modification processes. However, the initial and conditioned TPS_100_BC_0.02_SHMP_x_ specimens modified with SHMP contents higher than 10 phr had higher moisture contents than those of the initial and conditioned TPS_100_BC_0.02_SHMP_10_ specimens, respectively, since the excessive and unreacted SHMP molecules exhibit strong hygroscopicity.

New melting endotherms and diffraction peaks of V_H_-type crystals were found on DSC thermograms and WAXD patterns of TPS or TPS_100_BC_0.02_ specimens conditioned for 7 days, while no new melting endotherm or diffraction peak was found for TPS_100_BC_0.02_SHMP_x_ and/or (TPS_100_BC_0.02_SHMP_x_)_75_PLA_25_ specimens conditioned for less than 14 and 28 days, respectively. The rapid reduction in σ_f_ and I_s_ but increase in ɛ_f_ values of the conditioned TPS and TPS_100_BC_0.02_ specimens is apparently due to the abundant amounts of moisture absorbed during their conditioning processes, because the absorbed water molecules can effectively plasticize, soften and recrystallize starch molecules during their conditioning processes. The σ_f_ and I_s_ values of initial and conditioned TPS_100_BC_0.02_SHMP_x_ and (TPS_100_BC_0.02_SHMP_x_)_7__5_PLA_25_ specimens improved significantly and reached a maximal value as SHMP contents approached an optimal value at 10 phr, while their moisture content and ɛ_f_ values reduced to a minimal value, respectively, as SHMP contents approached 10 phr. Apparently, this is due to the best improved waterproof properties of the TPS_100_BC_0.02_SHMP_10_ and (TPS_100_BC_0.02_SHMP_10_)_7__5_PLA_25_ specimens modified with the optimal content of SHMP at 10 phr during their modification processes. In fact, after conditioning at 20 °C/50% RH for 56 days, the σ_f_, I_s_ and ɛ_f_ value of conditioned (TPS_100_BC_0.02_SHMP_10_)_75_PLA_25_ specimens remained at 19.5 MPa, 0.8 KJ/m^2^ and 13.1%, respectively, which are equivalent to about 52%, 50% and 3 times their initial σ_f_, I_s_ and ɛ_f_ values, respectively and 32.5 times, 8.9 times and 40% those of corresponding TPS_100_BC_0.02_ specimen conditioned for 56 days, respectively.

## References

[1] Nafchi A.M., Moradpour M., Saeidi M., Alias A.K. (2013). Thermoplastic starches: Properties, challenges, and prospects. Starch.

[2] Imberty A., Buléon A., Tran V., Péerez S. (1991). Recent advances in knowledge of starch structure. Starch.

[3] Cerclé C., Sarazin P., Favis B.D. (2013). High performance polyethylene/thermoplastic starch blends through controlled emulsification phenomena. Carbohydr. Polym..

[4] Mathew A.P., Dufresne A. (2002). Plasticized waxy maize starch: Effect of polyols and relative humidity on material properties. Biomacromolecules.

[5] Zhang Y.R., Wang X.L., Zhao G.M., Wang Y.Z. (2013). Influence of oxidized starch on the properties of thermoplastic starch. Carbohydr. Polym..

[6] Angellier H., Molina-Boisseau S., Dole P., Dufresne A. (2006). Thermoplastic starch-waxy maize starch nanocrystals nanocomposites. Biomacromolecules.

[7] Zhang Y.R., Zhang S.D., Wang X.L., Chen R.Y., Wang Y.Z. (2009). Effect of carbonyl content on the properties of thermoplastic oxidized starch. Carbohydr. Polym..

[8] Wang N., Yu J., Chang P.R., Ma X. (2008). Influence of formamide and water on the properties of thermoplastic starch/poly(lacticacid) blends. Carbohydr. Polym..

[9] Ma X.F., Yu J.G., Wan J.J. (2006). Urea and ethanolamine as amixed plasticizer for thermoplastic starch. Carbohydr. Polym..

[10] Daroz A.L., Zambom M.D., Curvelo A.A.S., Carvalho A.J.F. (2011). Thermoplastic starch modified during melt processing with organic acids: The effect of molar mass on thermal and mechanical properties. Ind. Crop. Prod..

[11] Kurosumi A., Sasaki C., Yamashita Y., Nakamura Y. (2009). Urea and ethanolamine as a mixed plasticizer for thermoplastic starch. Carbohydr. Polym..

[12] Yamanaka S., Watanabe K., Kitamura N., Iguchi M., Mitsuhashi S., Nishi Y., Uryu M. (1989). The structure and mechanical properties of sheets prepared from bacterial cellulose. J. Mater. Sci..

[13] Cristian J.G., Fernando G.T., Clara M.G., Omar P.T., Josep C.F., Juan M.P. (2009). Development of self-assembled bacterial cellulose-starch nanocomposites. Mater. Sci. Eng..

[14] Gandini A. (2008). Polymers from Renewable Resources: A challenge for the future of macromolecular materials. Macromolecules.

[15] Khaled E.T., Richard A.V., Joel J.P. (2007). Aspects of the preparation of starch microcellular foam particles crosslinked with glutaraldehyde using a solvent exchange technique. Carbohydr. Polym..

[16] Lee K.Y., Buldum G., Mantalaris A., Bismarck A. (2014). More than meets the eye in bacterial cellulose: Biosynthesis, bioprocessing, and applications in advanced fiber composites. Macromol. Biosci..

[17] Yeh J.T., Tsai C.C., Wang C.K., Shao J.W., Xiao M.Z., Chen S.C. (2014). Ultradrawing novel ultra-high molecular weight polyethylene fibers filled with bacterial cellulose nanofibers. Carbohydr. Polym..

[18] Tomasik P., Wang Y.J., Jane J.L. (1995). Facile route to anionic starches. Succinylation, maleination and phthalation of corn starch on extrusion. Starch.

[19] Hablot E., Dewasthale S., Zhao Y.J., Yang Z.G., Shi X.K., Graiver D., Narayan R. (2013). Reactive extrusion of glycerylated starch and starch–polyester graft copolymers. Eur. Polym. J..

[20] Zhou J., Ren L.L., Tong J., Xie L., Liu Z.Q. (2009). Surface esterification of corn starch films: Reaction with dodecenyl succinic anhydride. Carbohydr. Polym..

[21] Cova A., Sandoval A.J., Balsamo V., Mȕller A.J. (2010). The effect of hydrophobic modifications on the adsorption isotherms of cassava starch. Carbohydr. Polym..

[22] Van Soest J.J.G., Bezemer R.C., de Wit D., Vilegenthart J.F.G. (1996). Influence of glycerol on the melting of potato starch. Ind. Crop. Prod..

[23] Halley P.J., Smith R. (2005). Biodeegradable Polymers for Industrial Applications.

[24] Cheetham N.W.H., Tao L.P. (1998). Variation in crystalline type with amylose content in maize starch granules: An X-ray powder diffraction study. Carbonhydr. Polym..

[25] Perera C., Lu Z., Sell J., Jane J. (2001). Comparison of physicochemical properties and structures of sugary-2 cornstarch with normal and waxy cultivars. Cereal. Chem..

[26] Van Soest J.J.G., Vliegenthart J.F.G. (1997). Crystallinity in starch plastics: Consequences for material properties. Trends. Biotechnol..

[27] Kalichevsky M.T., Jaroszkiewcz E.M., Blanshard J.M.V. (1993). Study of the glass transition of amylopectin-sugar mixtures. Polymer.

[28] Noel T.R., Ring S.G., Whittam M.A. (1991). Kinetic aspects of the glass-transition behaviour of maltose—Water mixtures. Carbohydr. Res..

[29] Huang M.F., Yu J.G., Ma X.F. (2005). Ethanolamine as a novel plasticiser for thermoplastic starch. Polym. Degrad. Stabil..

[30] Choi H.M., Kim J.H., Shin S. (1999). Characterization of cotton fabrics treated with glyoxal and glutaraldehyde. J. Appl. Polym. Sci..

[31] Yamashita Y., Hirai N. (1966). Single crystals of amylose V complexes. II. Crystals with 71 helical configuration. J. Polym. Sci..

[32] Yamashita Y., Monobe K. (1971). Single crystals of amylose V complexes. III. Crystals with 81 helical configuration. J. Polym. Sci..

[33] Zobel H.F., French A.D., Hinckle M.E. (1967). X-ray diffraction of oriented amylose fibers. II. Structure of V amyloses. Biopolymers.

[34] Van Soest J.J.G., Knooren N.J. (1997). Influence of glycerol and water content on the structure and properties of extruded starch plastic sheets during aging. J. Appl. Polym. Sci..

[35] Kainuma K. (1988). The Biochemistry of Plants.

[36] Van Soest J.J.G., Hulleman S.H.D., de Wit D., Vliegenthart J.F.G. (1996). Changes in the mechanical properties of thermoplastic potato starch in relation with changes in B-type crystallinity. Carbohydr. Polym..

[37] Yu J.G., Wang N., Ma X.F. (2005). The effects of citric acid on the properties of thermoplastic starch plasticized by glycerol. Starch.

[38] Ma X.F., Chang P.R., Yu J.G., Stumborg M. (2009). Properties of biodegradable citric acid-modified granular starch/thermoplastic pea starch composites. Carbohydr. Polym..

[39] Bengtsson M., Koch K., Gatenholm P. (2003). Surface octanoylation of high-amylose potato starch films. Carbohydr. Polym..

[40] Carvalho A.J.F., Curvelo A.A.S., Gandini A. (2005). Surface chemical modification of thermoplastic starch: Reactions with isocyanates, epoxy functions and stearoyl chloride. Ind. Crop. Prod..

[41] Kulicke W.M., Aggour Y.A., Nottelmann H., Elsabee M.Z. (1989). Swelling and rheological studies of some starch hydrogels. Starch.

[42] Sagar A.D., Merrill E.W. (1995). Starch fragmentation during extrusion processing. Polymer.

[43] Kulicke W.M., Aggour Y.A., Elsabee M.Z. (1990). Preparation, Characterisation, and rheological behaviour of starch-sodium trimetaphosphate hydrogels. Starch.

[44] Zhou J., Zhang J., Ma Y.H., Tong J. (2008). Surface photo-crosslinking of corn starch sheets. Carbohydr. Polym..

[45] Jane J.L., Lim S., Paetau I., Spence K., Wang S. (1994). Polymers from Agricultural Coproducts.

[46] Woggum T., Sirivongpaisal P., Wittay T. (2014). Properties and characteristics of dual-modified rice starch based biodegradable films. Int. J. Biol. Macromol..

[47] Delval F., Crini G., Bertini S., Morin-Crini N., Vebrel J., Torri G. (2004). Characterization of crosslinked starch materials with spectroscopic techniques. J. Appl. Polym. Sci..

[48] Singh A.V., Nath L.K. (2012). Evaluation of acetylated moth bean starch as a carrier for controlled drug delivery. Int. J. Biol. Macromol..

[49] Kister G., Cassanas G., Vert M. (1998). Effects of morphology, conformation and configuration on the IR and Raman spectra of various poly(lactic acid)s. Polymer.

[50] Agarwal M., Koelling K.W., Chalmers J.J. (1998). Characterization of the degradation of polylactic acid polymer in a solid substrate environment. Biotechnol. Progr..

[51] Liu X.B., Zou Y.B., Li W., Cao G.P., Chen W.J. (2006). Kinetics of thermo-oxidative and thermal degradation of poly(d,l-lactide) (PDLLA) at processing temperature. Polym. Degrad. Stabil..

[52] Hsein-Chih H.W., Sarko A. (1978). The double-helical molecular structure of crystalline a-amylose. Carbohydr. Res..

[53] Hizukuri S. (1985). Relationship between the distribution of the chain length of amylopectin and the crystalline structure of starch granules. Carbohydr. Res..

[54] Ikada Y., Jamshidi K., Tsuji H., Hyon S.H. (1987). Stereocomplex formation between enantiomeric poly(lactides). Macromolecules.

